# Alcohol drinking patterns among high school students in Ethiopia: a cross-sectional study

**DOI:** 10.1186/1471-2458-12-213

**Published:** 2012-03-20

**Authors:** Ayalu A Reda, Asmamaw Moges, Berhanu Y Wondmagegn, Sibhatu Biadgilign

**Affiliations:** 1 Department of Public Health, College of Health Sciences, Haramaya University, P. O. Box 235 Harar, Ethiopia; 2 Department of Environmental Health Science, College of Health Sciences, Haramaya University, Harar, Ethiopia; 3 Department of Epidemiology and Biostatistics, College of Medical and Health Sciences, Jimma University, Jimma, Ethiopia

## Abstract

**Background:**

Alcohol use is an important risk factor for morbidity, mortality and social harm among adolescents. There is paucity of data on alcohol use among high school students in Ethiopia. This study aimed to determine the prevalence and factors associated with alcohol use among high school students in Ethiopia

**Methods:**

A cross-sectional study was conducted to assess the prevalence of alcohol use and its predictors among high school students in eastern Ethiopia in April 2010. A sample of students was taken from all schools based on their enrollment size. Prevalence estimates and their 95% confidence intervals were calculated. Logistic regression was performed to adjust and examine associations.

**Results:**

A total of 1721 students participated in the study. The mean age of the study population was 16.4 (SD 1.6) years. A total of 372 (22.2%; 95% CI 20.2 - 24.2%) students drink alcohol. Of these, 118 (31.7%) were females and 254 (68.3) males. Multivariate analysis indicated that males (OR 2.09; 95% CI 1.45-3.00), older age (OR 1.16; 95% CI 1.01-1.34), having friends who used alcohol (OR 10.09; 95% CI 6.84-14.89) and living with people who use alcohol (OR 2.77; 95% CI 1.89-4.07) increased the odds of drinking among students.

**Conclusion:**

There is a high level of alcohol use among high school students in the study area. Involvement of parents, health workers and school authorities are necessary to avert the problem. Specifically, their involvement in awareness campaigns and peer education training are important to encourage students to avoid alcohol use.

## Background

There is global concern about drinking trends among young people. Alcohol consumption is an important risk factor for morbidity, mortality and social harm worldwide [1-3] leading to 2.5 million deaths each year [4]. It is responsible for approximately 4% of the global burden of disease. This burden is higher in high income countries and among men, accounting for 11% of all male deaths in the World Health Organization (WHO) European region in 2004 [2]. Even though the problem is said to be increasing in the developing world, there are no sufficient data on alcohol use and its consequences in many developing countries [4,5].

The use of alcohol during the teenage and young adulthood years is a common phenomenon in many societies. For large numbers of youth it may signify psychological experimentation and risk taking [6]. Epidemiological studies indicate that a substantial proportion of alcohol users progress to problematic drinking or become alcohol dependent [7]. Alcohol consumption at a young age increases the risk of developing alcohol related problems later in life [8-11]. Among youth, drinking often coexists with other problem behaviors such as poor academic performance [12] and absenteeism [13] which may impair healthy development and successful transition from adolescence to adulthood [14].

A study conducted among high school adolescents in Ethiopia from 2001 to 2002 reported that about 8.9% drunk alcohol at least on a weekly bases [15], where as other reports among students in southern Ethiopia and a private school in Addis Ababa found a prevalence of 57.7% and 19.2% respectively [5]. In other sub-Saharan African countries like Kenya ever drinking prevalence of up to 15% were found among secondary school students [16], where some private universities had rates as high as 84% [17]. A study from South Africa also reported an alcohol use prevalence of 39.1% among high school adolescents [18].

As in other developing countries, the distribution and consumption of substances including alcohol are not sufficiently studied in Ethiopia [5,19]. Alcohol consumption is not legally prohibited in Ethiopia and there are no age limits on alcohol drinking [19]. Culturally, it is consumed in social gatherings and among friends as a pass time activity and relaxation experience [19]. Description of the relationship between alcohol use and other important variables would usefully guide national policy and decision making on consumption patterns and how its impact could be mitigated. The main aim of this study was to determine the prevalence and factors associated with alcohol use among high school students in eastern Ethiopia.

## Methods

### Study setting and context

The study was conducted in Harar town located 525 km east of the capital city, Addis Ababa. Harari Regional State is one of the nine regions in Ethiopia and Harar town is its capital city. The town has nine high schools from grades 9 to 12 attended by adolescents. The total number of students enrolled in these schools in the academic year at the time of the study was 6523.

### Study design and sample size

A descriptive cross-sectional study was conducted in April 2010 among high school students to assess the prevalence of alcohol use and its predictors. The sample size was calculated using the formula for estimation of a single proportion [20], n = z^2^*p(1-p)/d^2^. Where the z value is taken as 1.96; p, proportion of alcohol drinking, was assumed to be 36%; and d, the margin of error of estimation, was assumed to be 3% or 0.03. This provided a sample size of 990. And with a design effect of 2 and a 10% non-response rate, the final the sample size was 1890 students. Finally, a total of 1890 students, representing 29% of the study population, were surveyed. All schools were included in the study and a proportional stratified sampling technique was employed to obtain study units. A proportional sample of students were selected from each school and grades based on their enrollment size at the time of the study. Within grades, all students in the selected classes participated regardless of their age.

### Data collection procedure

A self administered structured questionnaire consisting of open and closed ended questions was developed based on a review of the literature and by adaptation of the Youth Risk Behavior Survey (YRBS) questionnaire developed by the U.S. Centers for Disease Control and Prevention (CDC) [21,22]. The anonymous questionnaire covers socio-demographic factors in addition to alcohol drinking habits. The English version of the questionnaire was translated into Amharic, the official language of the study area, by a panel of experts fluent in the language. It was then translated back into English by another person to ensure consistency with the English language questionnaire. Pre-testing of the questionnaire was undertaken in 5 percent of the sample size (95 students in schools outside but close to the study area) in similar areas before the actual data collection took place and corrections were made thereafter to improve the clarity of some items. Cultural equivalence was also sought in the adoption. For instance items on cannabis and violence were dropped since cocaine use is less known in the study area and that violence is not the objective of the study. The final version of the Amharic language questionnaire was used for the data collection. The participants anonymously responded to the items on the questionnaire using pen or pencils.

### Variables

The dependent variables were alcohol use and its frequency. The independent variables included age, sex, religion, education/grade level, practice of alcohol use among family and friends, money spent on alcohol, previous exposure to drugs.

### Operational definition

A student was considered as ever drinker if s/he responds yes to the question 'Have you ever drunk alcohol in your life?' Then follow up questions were employed to collect information such as drinking in the past 30 days and frequency of drinking. Current alcohol use is defined as use of alcohol at least once during the past 30 days before the survey.

### Statistical analysis

The data were coded, entered, cleaned, and analyzed using IBM^® ^SPSS^® ^Statistics, version 15 for Windows. Descriptive statistics were conducted using frequencies and proportions. Bivariate and multivariate analyses were carried out using logistic regression to examine the relationship between the outcome variable of alcohol use and selected predictors. The multivariate logistic regression was conducted using simultaneous entry of independent variables. Outliers were inspected and multicollinearity among variables was examined using simultaneous entry linear regression. Adjusted and unadjusted odds ratios (OR) and their 95% confidence intervals (CI) were used as indicators of strength of association. A p-value of 0.05 or less was used as the cut-off level for statistical significance.

### Ethics statement

Ethical clearance was obtained from Institutional Research Ethics Review Committee of Haramaya University, College of Health Sciences (Date, March 04, 2010). A letter of cooperation was written from Haramaya University and further approval was obtained from the regional health bureau and then the surveyed schools. The objective of the study was explained to the study participants. They were briefed about the confidentiality of their response and the importance of providing correct and accurate information, and that participation was voluntary. All participants included in the study have provided written consent.

## Results

### Study population characteristics

A total of 1721 students participated in the study with a response rate of 91.1%. Of these 856 (50.1%) were males and the rest 851 (49.9%) females, with a mean (SD) age of 16.4 (1.6) years. The majority, 839 (48.9%) of the students were 9^th ^graders. The majority (92.5%) of the respondents were living with their parents at the time of the study (Tables 1 and 2).

**Table 1:** Socio-demographic characteristics of the sampled high school students in eastern Ethiopia (n = 1721)

Variables	Frequency^¥^	Percent
Sex		

Female	851	49.9

Male	856	50.1

Age		

15-19	1085	63.5

20-25	622	36.4

Religion		

Orthodox	901	52.8

Protestant	36	2.1

Catholic	549	32.2

Muslim	191	11.2

Others	29	1.7

Grade		

9^th^	839	48.9

10^th^	394	23.0

11^th^	253	14.7

12^th^	230	13.4

Living with		

Parents	1288	92.5

Friends	27	1.9

Relatives	43	3.1

Alone	34	2.4

^¥^Based on the total number of respondents to each item

**Table 2:** Characteristics of alcohol ever drinkers among the sampled high school students (n = 372)

Factors	Ever drinkers' frequency (%)^§, ¥^
Sex	

Male	254 (68.3)

Female	118 (31.7)

Age, Mean(SD)	17.5 (2.5)

Grade	

9^th^	150 (40.2)

10^th^	74 (19.8)

11^th^	65 (17.4)

12^th^	84 (23.3)

Religion	

Orthodox Christian	278 (74.7)

Protestant	20 (5.4)

Catholic	43 (11.6)

Muslim	27 (7.3)

Others	4 (1.1)

Have friends who drink alcohol	

Yes	302 (81.8)

No	67 (18.2)

Living with people who smoke cigarettes	

Yes	278 (75.3)

No	91 (24.7)

Have you drunk in the past 30 days?	

Yes	179 (10.4)

No	1542 (89.6)

Ever drunk alcohol as well as chewed *khat^§^*	

Yes	186 (50.0)

No	186 (50.0)

Ever drunk alcohol as well as smoked	

Yes	114 (30.6)

No	258 (69.4)

^§^Number (percent) unless indicated otherwise

^§^*Khat *is a stimulant leaf extensively consumed in east Africa and the Arabian Peninsula that has amphetamine and could lead to addiction and other negative consequences

^¥^Based on the total number of respondents to each item

### Alcohol use of the study subjects

A total of 372 (22.2%; 95% CI 20.2 - 24.2%) respondents had ever drank alcohol, 179 (10.4%; 95% CI 9.0-11.8%) have drank alcohol in the past 30 days. More males (254; 68.3%) than females (118; 31.7%) were ever drinkers. The mean age of drinkers was 17.5 years, 17.3 for males and 17.8 for females. There was no difference in mean age of drinking by sex (t = 1.909, p = 0.06). Two hundred and ninety six (79.5%) of the respondents drank alcohol in bars and restaurants, while 62 (16.7%) drank at home. The majority of the drinkers (60.5%) drank alcohol with their friends and 119 (32.1%) consumed with their parents. Thirty eight percent of the participants were living with people who drank alcohol (living arrangements include 46.0% with father or mother; 17.2%, brothers or sisters; 16.6%, with relatives; and 14.3%, with friends). About 34% of the participants had friends who drank alcohol. Concerning the source of money for buying alcohol, 266 (71.6%) of the drinkers got money from their parents and 55 (14.9%) from friends. Drinkers spent an average of 65.2 Ethiopian birr ($4.1) per week for alcohol. About 31% (114) and 46% (186) of the students were also smokers and *Khat *chewers respectively on top of alcohol drinking. Further details are provided in the Table 2.

### Determinants of drinking alcohol, multivariate results

Logistic regression analysis was performed to identify the effect of each variable on drinking alcohol. Multivariate analysis of the dependent variable (alcohol drinking) with other outcome variables revealed that sex, age, having friends who drink alcohol and living with people who consume alcohol have strong association with drinking alcohol among the students. Though the bivariate analysis showed significant difference in drinking alcohol by grade level and religion, this difference disappeared in the multivariate analysis (Table 3).

**Table 3:** Logistic regression estimates of independent predictors of alcohol drinking among sampled high school students in eastern Ethiopia (n = 1721)^§^

Explanatory variable	Unadjusted OR (95% CI)	p-value	Adjusted OR (95% CI)	p-value
Sex				

Male	2.67 (2.09-3.41)	< 0.010	2.09 (1.45-3.00)	< 0.001

Female	1.0		1.0	

Age	1.32 (1.22-1.42)	< 0.010	1.16 (1.01-1.34)	0.030

Grade				

9^th^	1.00		1.00	

10^th^	0.84 (0.65-1.21)	0.438	1.30 (0.87-1.95)	0.204

11^th^	0.64 (0.46-0.89)	< 0.010	0.98 (0.64-1.51)	0.937

12^th^	0.38 (0.28-0.53)	< 0.001	0.77 (0.51-1.17)	0.216

Having friends who drink alcohol				

Yes	17.56 (13.04-23.65)	< 0.010	10.09 (6.84-14.89)	< 0.001

No	1.0		1.0	

Living with people who drink alcohol				

Yes	7.93 (6.08-10.36)	P < 0.010	2.77 (1.89-4.07)	< 0.001

No	1.0		1.0	

^§^There were no outliers detected (standard deviations outside ± 3) nor multicollinearity

## Discussion

There is a growing concern regarding the increase in preventable harms attributed to adolescent alcohol consumption [23]. Such concern has prompted medical and research debate regarding the delaying of adolescent alcohol consumption for as long as possible [7,8,24]. This study tried to investigate alcohol use among high school adolescents in eastern Ethiopia.

The findings indicate an alcohol use prevalence of 22.2% (95% CI 20.2 - 24.2%) among the sampled high school students. About 10% (95% CI 9.0-11.8%) have drank in the past 30 days. We have also identified that male gender (OR 2.09; 95% CI 1.45-3.00), older age (OR 1.16; 95% CI 1.01-1.34), having friends who drink alcohol (OR 10.09; 95% CI 6.84-14.89) or living with people who use alcohol (OR 2.77; 95% CI 1.89-4.07) as strong predictors of drinking among the students.

Our finding of ever and past month alcohol drinking of 22.2% and 10.4% respectively seem to be lower than the report from Addis Ababa which indicated alcohol drinking prevalence of 45.7% and 26.5% in the same order [25]. The main explanation for this is probably that Harar town is more cultural and value oriented than those studied in Addis Ababa. Also the fact that there is less exposure to alcohol advertising compared to Addis Ababa where billboards and FM radio advertisements are abound and could contribute to the lower drinking level in this study [26,27]. It is also lower than 41.8% past 30 days drinking by high school students in the USA [28] and 51.9% ever-drinking prevalence of alcohol use reported among secondary school students in Kisumu town, Kenya [29]. Similarly, a South African study reported an alcohol use prevalence of 39.1% among high school adolescents [18]. Other studies among high school students in Africa also report similar finding of a high prevalence rates of alcohol use, ranging from 15% to 57.9% [16,18,30]. The primary reason for the comparatively lower alcohol drinking level in this study could be lack of access due to cultural and economic reasons. Despite this, the level of drinking in this study is far from normal and should be a cause for concern as it is a dangerous precedent for other risky behaviors both during adolescence and in adult life [24]. Already, 30 percent of ever drinkers have also smoked a cigarette. Furthermore there are no assurances that the observed level of drinking could not progress to high levels of drinking over time and multi-substance use.

In this study, the average age at which the respondents started using alcohol was 17.5 years. On average, both sexes seem to start drinking beyond the high risk age of 14 years, which is associated with high risk for alcohol abuse and dependence in later life according to studies on sample populations from the USA [24]. However, a recent systematic review indicates that later adolescence drinking could progress into late adulthood drinking habits, and it was also associated with suicide, car crashes and mental and social problems [31]. Currently, a legal age limit for drinking as well as other important ordinances that ban drinking in certain places and advertising that tempt adolescents do not exist in the country, probably indicating the little attention given to adolescent drinking [19]. This is understandable given the infectious disease focus of health authorities in developing countries.

Our findings indicate that males have higher odds of drinking than females (2.09; 95% CI 1.45-3.00). This is a common finding in substance use studies where males use substances more and show higher tendency for dependence than females. Apart from measurement and reporting issues [32,33], it could mainly be explained by higher exposure opportunities due to reasons such as psychological, family and social factors [32,34,35]. The findings also indicate that having friends who drink alcohol and living with people who drink alcohol are very strong predictors of alcohol use among the sample studied. This is in line with the consistent findings in the literature concerning peer pressure, influence of social groups and the suggestibility of adolescents toward alcohol drinking as well as other substances [7,36-38]. Hence, this calls for awareness campaigns that have more focus on social groups and peers.

This study has limitations. One of them is the use of self-reporting methods. Self-reporting is prone to recall bias and under reporting due to social desirability bias. As a result the reported data are most likely an underestimation of true levels of drinking. However, we could not also exclude the possibility of exaggeration [39]. The other important consideration is that non-respondents might have higher levels of drinking compared to respondents. This is because one of the reasons of non-response in this study is absence from class which could be due to substance related delinquency. Furthermore, close to half of the sample is composed of ninth graders. The trend of decreasing student numbers in subsequent classes might be due to a range of underlying factors influencing school attendance, the role of risk behaviors such as substance use cannot be ruled out.

## Conclusions

In conclusion, our findings indicate that there are high levels of alcohol drinking among the sampled high school students. Parents, as well as school and health authorities need to work more toward awareness creation about the hazards of alcohol drinking. We also call upon policy makers to put in place ordinances for adolescent drinking.

## Competing interests

The authors declare that they have no competing interests.

## Authors' contributions

AAR, AM and BY designed and compiled data collection instruments. AAR, AM and BY wrote the proposal. AM and BY have involved significantly in data collection. AAR and AM involved in data analysis. AAR and SB have contributed significantly to the writing of the manuscript. All authors read and approved the final manuscript.

## Pre-publication history

The pre-publication history for this paper can be accessed here:

http://www.biomedcentral.com/1471-2458/12/213/prepub

## References

[B1] Rehm J Room R Monteiro M Gmel G Graham K Rehn N Sempos CT Jernigan D Alcohol as a risk factor for global burden of disease Eur Addict Res 2003 9 4 157 164 10.1159/000072222 12970584

[B2] Rehm J Mathers C Popova S Thavorncharoensap M Teerawattananon Y Patra J Global burden of disease and injury and economic cost attributable to alcohol use and alcohol-use disorders Lancet 2009 373 9682 2223 2233 10.1016/S0140-6736(09)60746-7 19560604

[B3] Beaglehole R Bonita R Alcohol: a global health priority Lancet 2009 373 9682 2173 2174 10.1016/S0140-6736(09)61168-5 19560583

[B4] WHO Global status report on alcohol. Mental health and Substance Abuse 2011 Geneva: World Health Organization

[B5] Alem A Kebede D Berhane Y, Haile-Mariam Damen, Kloos H Alcohol and substance abuse Epidemiology and ecology of health and diseases in Ethiopia 2006 Addis Ababa: Ethiopian Public Health Association (EPHA) 807 827

[B6] Shedler J Block J Adolescent drug use and psychological health. A longitudinal inquiry Am Psychol 1990 45 5 612 630 2350080 10.1037//0003-066x.45.5.612

[B7] Merline A Jager J Schulenberg JE Adolescent risk factors for adult alcohol use and abuse: stability and change of predictive value across early and middle adulthood Addiction 2008 103 Suppl 1 84 99 18426542 10.1111/j.1360-0443.2008.02178.x PMC2649657

[B8] Hingson RW Heeren T Winter MR Age at drinking onset and alcohol dependence: Age at onset, duration, and severity Arch Pediatr Adolesc Med 2006 160 739 746 10.1001/archpedi.160.7.739 16818840

[B9] Grant BF Stinson FS Harford TC Age at onset of alcohol use and DSM-IV alcohol abuse and dependence: a 12-year follow-up J Subst Abuse 2001 13 4 493 504 10.1016/S0899-3289(01)00096-7 11775078

[B10] Moore E Coffey C Carlin JB Alati R Patton GC Assessing alcohol guidelines in teenagers: results from a 10-year prospective study Aust N Z J Public Health 2009 33 2 154 159 10.1111/j.1753-6405.2009.00363.x 19413860

[B11] Bonomo YA Bowes G Coffey C Carlin JB Patton GC Teenage drinking and the onset of alcohol dependence: a cohort study over seven years Addiction 2004 99 12 1520 1528 10.1111/j.1360-0443.2004.00846.x 15585043

[B12] Mason WA Windle M Family, religious, school and peer influences on adolescent alcohol use: a longitudinal study J Stud Alcohol 2001 62 1 44 53 11271963 10.15288/jsa.2001.62.44

[B13] Gruber E DiClemente RJ Anderson MM Lodico M Early drinking onset and its association with alcohol use and problem behavior in late adolescence Prev Med 1996 25 3 293 300 10.1006/pmed.1996.0059 8781007

[B14] Schulenberg J O'Malley PM Bachman JG Wadsworth KN Johnston LD Getting drunk and growing up: trajectories of frequent binge drinking during the transition to young adulthood J Stud Alcohol 1996 57 3 289 304 8709588 10.15288/jsa.1996.57.289

[B15] Kebede D Alem A Mitike G Enquselassie F Berhane F Abebe Y Ayele R Lemma W Assefa T Gebremichael T Khat and alcohol use and risky sex behaviour among in-school and out-of-school youth in Ethiopia BMC Public Health 2005 5 109 10.1186/1471-2458-5-109 16225665 PMC1274331

[B16] Kuria MW Drug abuse among urban as compared to rural secondary schools students in Kenya: a short communication East Afr Med J 1996 73 5 339 8756042

[B17] Odek-Ogunde M Pande-Leak D Prevalence of substance use among students in a Kenyan University: a preliminary report East Afr Med J 1999 76 6 301 306 10750515

[B18] Madu SN Matla MQ Illicit drug use, cigarette smoking and alcohol drinking behaviour among a sample of high school adolescents in the Pietersburg area of the Northern Province, South Africa J Adolesc 2003 26 1 121 136 10.1016/S0140-1971(02)00120-3 12550825

[B19] EPHA Teshome T Legal aspects of substance and alcohol abuse in Ethiopia: Executive summary22nd Annual Conference of the Ethiopian Public Health Association 2011 Addis Ababa: Ethiopian Public Health Association

[B20] Kelsey JL Whittemore AS Evans AS Thompson WD Methods of sampling and estimation of sample size Methods in observational epidemiology 1996 New York and Oxford: Oxford University Press 311 340

[B21] Kann L The Youth Risk Behavior Surveillance System: Measuring health-risk behavior Am J Health Behav 2001 25 272 277 10.5993/AJHB.25.3.14 11322626

[B22] Kolbe LJ Kann L Collings JL Overview of the youth risk behavior surveillance system Public Health Reports 1993 108 Suppl1 2 10 8210269 PMC1403301

[B23] Livingston M Recent trends in risky alcohol consumption and related harm among young people in Victoria, Australia Aust N Z J Public Health 2008 32 3 266 271 10.1111/j.1753-6405.2008.00227.x 18578827

[B24] DeWit DJ Adlaf EM Offord DR Ogborne AC Age at first alcohol use: a risk factor for the development of alcohol disorders Am J Psychiatry 2000 157 5 745 750 10.1176/appi.ajp.157.5.745 10784467

[B25] Teshome D Prevalence of substance use and its determinants among highschool students in Addis Ababa22nd Conference of the Ethiopian Public Health Association. Volume Abstract 5 2011 Addis Ababa: EPHA 15 16

[B26] Anderson P de Bruijn A Angus K Gordon R Hastings G Impact of alcohol advertising and media exposure on adolescent alcohol use: a systematic review of longitudinal studies Alcohol Alcohol 2009 44 3 229 243 19144976 10.1093/alcalc/agn115

[B27] Ellickson PL Collins RL Hambarsoomians K McCaffrey DF Does alcohol advertising promote adolescent drinking? Results from a longitudinal assessment Addiction 2005 100 2 235 246 10.1111/j.1360-0443.2005.00974.x 15679753

[B28] Eaton DK Kann L Kinchen S Shanklin S Ross J Hawkins J Harris WA Lowry R McManus T Chyen D Youth risk behavior surveillance - United States, 2009 MMWR Surveill Summ 2010 59 5 1 142 20520591

[B29] Atwoli L Mungla PA Ndung'u MN Kinoti KC Ogot EM Prevalence of substance use among college students in Eldoret, western Kenya BMC Psychiatry 2011 11 34 10.1186/1471-244X-11-34 21356035 PMC3053226

[B30] Otieno AO Ofulla AVO Drug abuse in Kisumu town western Kenya Afr J Food, Agric, Nutr Dev 2009 2009 3 846 858

[B31] McCambridge J McAlaney J Rowe R Adult consequences of late adolescent alcohol consumption: A systematic review of cohort studies PLoS Med 2011 8 2 e1000413 10.1371/journal.pmed.1000413 21346802 PMC3035611

[B32] Dawson DA Archer L Gender differences in alcohol consumption: effects of measurement Br J Addict 1992 87 1 119 123 10.1111/j.1360-0443.1992.tb01909.x 1543934

[B33] Dawson DA Methodological issues in measuring alcohol use Alcohol Res Health 2003 27 1 18 29 15301397 PMC6676704

[B34] Kullgren G Alibusa S Birabwa-Oketcho H Problem drinking among patients attending primary healthcare units in Kampala, Uganda Afr J Psychiatry (Johannesbg) 2009 12 1 52 58 19517048 10.4314/ajpsy.v12i1.30279

[B35] Nielsen MF Resnick CA Acuda SW Alcoholism among outpatients of a rural district general hospital in Kenya Br J Addict 1989 84 11 1343 1351 10.1111/j.1360-0443.1989.tb00736.x 2597810

[B36] Mundt MP The impact of peer social networks on adolescent alcohol use initiation Academic Pediatrics 2011 11 5 414 421 10.1016/j.acap.2011.05.005 21795133 PMC3170443

[B37] Newcomb MD Psychosocial predictors and consequences of drug use: a developmental perspective within a prospective study J Addict Dis 1997 16 1 51 89 10.1300/J069v16n01_05 9046445

[B38] Almeida-Filho N Lessa I Magalh es L Araujo MJ Aquino E Kawachi I James SA Alcohol drinking patterns by gender, ethnicity, and social class in Bahia, Brazil Rev Saude Publica 2004 38 1 45 54 14963541 10.1590/s0034-89102004000100007

[B39] Tourangeau R Yan T Sensitive questions in surveys Psychol Bull 2007 133 5 859 883 17723033 10.1037/0033-2909.133.5.859

